# Sulphur metabolism and cellulase gene expression are connected processes in the filamentous fungus *Hypocrea jecorina *(anamorph *Trichoderma reesei*)

**DOI:** 10.1186/1471-2180-8-174

**Published:** 2008-10-08

**Authors:** Gabriela Gremel, Marcel Dorrer, Monika Schmoll

**Affiliations:** 1 Research Area of Gene Technology and Applied Biochemistry, Institute of Chemical Engineering, Vienna University of Technology, Getreidemarkt 9/1665, A-1060 Wien, Austria

## Abstract

**Background:**

Sulphur compounds like cysteine, methionine and S-adenosylmethionine are essential for the viability of most cells. Thus many organisms have developed a complex regulatory circuit that governs the expression of enzymes involved in sulphur assimilation and metabolism. In the filamentous fungus *Hypocrea jecorina *(anamorph *Trichoderma reesei*) little is known about the participants in this circuit.

**Results:**

Analyses of proteins binding to the cellulase activating element (CAE) within the promotor of the cellobiohydrolase *cbh2 *gene led to the identification of a putative E3 ubiquitin ligase protein named LIMPET (LIM1), which is an orthologue of the sulphur regulators SCON-2 of *Neurospora crassa *and Met30p of *Saccharomyces cerevisiae*. Transcription of *lim1 *is specifically up-regulated upon sulphur limitation and responds to cellulase inducing conditions. In addition, light dependent stimulation/shut down of cellulase gene transcription by methionine in the presence of sulphate was observed. Further, *lim1 *transcriptionally reacts to a switch from constant darkness to constant light and is subject to regulation by the light regulatory protein ENVOY. Thus *lim1*, despite its function in sulphur metabolite repression, responds both to light as well as sulphur- and carbon source. Upon growth on cellulose, the uptake of sulphate is dependent on the light status and essential for growth in light. Unlike other fungi, growth of *H. jecorina *is not inhibited by selenate under low sulphur conditions, suggesting altered regulation of sulphur metabolism. Phylogenetic analysis of the five sulphate permeases found in the genome of *H. jecorina *revealed that the predominantly mycelial sulphate permease is lacking, thus supporting this hypothesis.

**Conclusion:**

Our data indicate that the significance of the sulphate/methionine-related signal with respect to cellulase gene expression is dependent on the light status and reaches beyond detection of sulphur availability.

## Background

Gene transcription in filamentous fungi is influenced by a variety of external stimuli. Adaptation to nutrient conditions is one of them. Many sulphur compounds, especially cysteine, methionine and S-adenosylmethionine are essential for the viability of most cells [1]. Thus, many organisms have developed a complex regulatory circuit that governs the expression of enzymes involved in sulphur assimilation and metabolism. Appropriate mechanisms have been discussed in the filamentous fungi *Aspergillus nidulans *and *Neurospora crassa *as well as the yeast *Saccharomyces cerevisiae *(reviewed by [1]). *Neurospora crassa *CYS3 [2] and *Saccharomyces cerevisiae *Met4 [3] were described as positive-acting regulators of an entire set of genes involved in sulphur metabolism. SCONB in *Aspergillus nidulans *[4,5], SCON2 in *Neurospora crassa *[6,7] and Met30 in *Saccharomyces cerevisiae *[8] on the other hand were identified as negative regulators. In recent years, the regulatory machinery of sulphur metabolism has been shown to be embedded in a more complex network, which also involves response to heavy metals [9] and is influenced by the supply of carbon and nitrogen in plants [10]. The SCF-ubiquitin-ligase complex SCF^(*Met*30) ^participates in cadmium-induced regulation of the transcription factor Met4 [11] and thereby initiates a system wide cellular response. This SCF-ubiquitin ligase complex is also presumed to contribute to the detoxification of cadmium by increasing the glutathion levels needed for complexing this toxic heavy metal [12,13]. A similar mechanism is likely to be at work for detoxification of arsenate [14] and selenate [15].

The filamentous fungus *Hypocrea jecorina *(anamorph *Trichoderma reesei*) is to date the most important industrial producer of cellulases [16-18]. Nevertheless, the genome of this fungus comprises a lower number of genes encoding these degradative enzymes than found in other filamentous fungi [19]. The importance of cellulases as indispensable reagents in industries like the pulp and paper industry, detergent industry or clothing industry gives rise to attempts for elucidating the mechanisms lying behind cellulose signalling to cellulase gene expression in *H. jecorina*. Remarkable effort had thus been put into elucidation of the mechanisms involved in regulation of cellulase gene expression. Besides cellulose, also lactose and sophorose promote cellulase gene expression, while glycerol does neither promote nor prevent it. Formation of cellulases is regulated at the transcriptional level, and the expression of the different cellulase genes has been reported to be coordinate [20]. However, little is known still about how the cellulose signal from outside the cell is transduced to initiate production of the appropriate hydrolytic enzymes. The first signal transduction protein to be identified in the respective signalling pathway was the light regulatory protein ENVOY [21], which also lead to the finding that cellulase gene expression is modulated by light. Subsequent analyses revealed that this protein influences several regulatory pathways in *H. jecorina *and that its function is not limited to light response [22].

Studies on the promotors of the cellulase genes provide a thorough basis to climb up the signal transduction cascade starting from the target: Using cell-free extracts from sophorose-induced and non-induced mycelia as well as various fragments of the promotor sequence of the *H. jecorina *cellobiohydrolase *cbh2*, the nucleotide sequence 5'-ATTGGGTAATA-3' was found to bind different protein complexes in electrophoretic mobility shift assays (EMSA) under the respective conditions [23]. The mentioned sequence was named *cbh2*-activating element CAE. The presence of an intact copy of this motif was shown to be required for protein binding and wild-type levels of *cbh2 *transcript [23]. While the HAP2/3/5 complex of *H. jecorina *was found to bind to the CCAAT-box of the cellulase activating element CAE within the *cbh2 *promoter [24] the factor(s) binding to the second part of the motif therein, which shows an even stronger regulatory effect (50% decrease in transcription efficiency upon mutation to 5' ATTGGGT**TT**TA 3') than the CCAAT-box, have not been described.

The transcriptional regulation of cellulase gene expression has been subject to extensive research [20,25], and among the regulatory factors of this process, ACE2 [26], and XYR1 [27,28] have been shown to bind to sequence motifs related yet not similar to CAE in the promotors of *cbh1 *(ACE2; 5' GGCTAATAA 3') or *xyn1 *(XYR1; 5' GGCTAA 3'). Despite the fact that their binding to CAE within the *cbh2 *promotor has not been proven yet, they are likely to contact this motif due to their involvement in regulation of *cbh2 *expression.

Our study was aimed at a more detailed understanding of cellulase gene regulation at the promotor level. Application of the Yeast One Hybrid System should identify the regulatory factor(s) binding to CAE. Surprisingly, we found a putative E3 ubiquitin ligase, the homologues of which are involved in regulation of sulphur metabolism, to bind to this regulatory sequence. This result led to the hypothesis that sulphur signaling/metabolism and regulation could be interconnected processes. In order to test this hypothesis we used different cultivation conditions appropriate to delineate the involvement of this E3 ubiquitin ligase in both processes and more importantly the role of the sulphur source with respect to cellulose utilization in *H. jecorina *in general. We show that the respective gene not only responds to cellulase inducing conditions and to light, but also – as expected – to a decrease in sulphur availability. Since methionine seems to have a specific function in regulation of cellulase gene expression under defined sulphur conditions, we tested its effect under conditions commonly used to analyse this process. Indeed, the strong light-dependent effect of increased methionine levels seen under defined sulphur conditions also occurs under these conditions i. e. in the presence of sulphate and peptone. Moreover, we detected a role of sulphate in cellulose utilization, but not glucose utilization in light. Subsequent screening of the *H. jecorina *genome for the presence of sulphate permeases revealed the lack of a homologue to the *N. crassa *predominantly mycelial sulphate permease CYS-14.

## Methods

### Microbial strains and culture conditions

*H. jecorina *(*T. reesei*) strain QM9414 (ATCC 26921), which is a second generation high cellulase producing mutant strain of wild-type QM6a and is commonly used for studies on this fungus representing wild-type conditions as well as the recombinant strain *env1*^PAS-^, which is lacking the PAS domain of the light regulatory protein ENVOY [21] were used throughout this study. *H. jecorina *was grown in submerged culture at 28°C in 1-liter Erlenmeyer flasks on a rotary shaker (200 rpm) in 200 ml of Mandels Andreotti minimal medium [29]. 1% (w/v) of the carbon sources indicated with the respective experiments was used. 10^8 ^conidia/l (final concentration) were used as inoculum. The replacement technique described by Sternberg and Mandels [30] was applied for inducing cellulase formation in mycelia by 1.5 mM sophorose (final concentration), as described previously [23,31].

The medium for cultivation under sulphur limitation conditions contained the respective chlorides instead of the sulphates used in conventional Mandels Andreotti medium and was prepared as follows: The modified trace element solution contained 0.243 g/l FeCl_3_·6H_2_O (0.90 mM), 0.050 g/l MnCl_2_·2H_2_O (0.31 mM), 0.033 g/l ZnCl_2 _(0.24 mM) and 0.100 g/l CoCl_2_·6H_2_O (0.42 mM) and the pH was adjusted to 2.0 with hydrochloric acid. The mineral salt solution contained 2.27 g/l NH_4_Cl (42 mM), 4.00 g/l KH_2_PO_4 _(29 mM), 0.50 g/l MgCl_2_·6H_2_O (2 mM), 0.80 g/l CaCl_2_·2H_2_O (5 mM) and 0.6 g/l urea (10 mM). A 0.1 M phosphate buffer was prepared using a solution with 35.6 g/l Na_2_HPO_4_·2H_2_O (0.2 M) and 0.2 M citric acid was added to adjust a pH of 5.0 (all chemicals by Merck, Germany). The culture medium was prepared combining 500 ml mineral salt solution, 480 ml phosphate buffer and 20 ml trace element solution.

As sulphur source, the following three concentrations of L-methionine (Sigma-Aldrich, St. Louis, USA) were used: 0.05 mM, 0.25 mM and 5 mM corresponding to limiting, low and high sulphur concentration, respectively [5].

Yeast strain YM4271 (MATa, *ura*3–52, *his*3–200, *ade*2–101, *lys*2–801, *leu*2–3, 112, *trp*1–903, *tyr*1–501, *gal4*-Δ512, *gal80*-Δ538, *ade5*::*hisG *[32,33] was used as host strain for the One Hybrid System.

*E. coli *JM109 [34] was used for the propagation of vector molecules and DNA manipulations; *E. coli *ER1647 (Novagen, Madison, WI) was used for plating, titering and screening of the λ Blue Star gene library; and *E. coli *strain BM25.8 (Novagen), which is lysogenic for λ phages, was used for automatic subcloning. The latter two strains were grown at 37°C on LB medium supplemented with 0.2% (w/v) maltose and 10 mM MgSO_4 _for propagation of λ-phages. *E. coli *BL21 (DE3) (Stratagene, La Jolla, CA) was used for the expression of GST fusion proteins according to the recommendations of the manufacturers.

### Nucleic acid isolation and blotting

*H. jecorina *DNA was isolated as described previously [31]. Yeast and *E. coli *DNA was isolated as described by Sambrook et al[35]. Total RNA was isolated by the guanidinium/phenol procedure [36]. Northern blotting was performed as described previously [31,35]. For transcript analysis of *lim1 *the insert of pOHS125 was excised and used for hybridization. Probes for analysis of transcription of *env1*, *cbh1*, *cbh2 *and 18S rRNA were prepared by PCR. Densitometric scanning was done using the BIO-RAD (Hercules, USA) GS-800 calibrated densitometer and the BIO-RAD Quantity One software for different exposures of the respective film.

### Construction of Reporter Plasmids and Yeast strains for One-hybrid screening

Complementary oligonucleotides with three copies of the mutated CAE motif (oneH(F) 5' AATTCTCTTTA**AA**GG**GTAATA**TACAGCCATCTTTA**AA**GG**GTAATA**TACAGCCATCTTTA**AA**GG**GTAATA**TACAGCCAT 3') containing the functional (GG)**GTAATA**-sequence and a non-functional CC**AA**T box [23] corresponding to positions – 253 to – 220 of the *cbh2 *promoter were cloned into plasmids pHISi and pLacZi. For the construction of a negative control plasmid also the (GG)GTAATA sequence was mutated to (GG)GTTTTA in the oligonucleotide to abolish binding of the respective proteins [23]. The resulting plasmids were designated pHISi-CAE and pLacZi-CAE or pHISi-mCAE, respectively.

A reporter strain HIS-LACZ-CAE for the transformation of the cDNA library was constructed by integration of pHISi-CAE and pLacZi-CAE into yeast strain YM4271. The negative control strain HIS-mCAE was obtained by integration of pHISi-mCAE into yeast strain YM4271. Plasmids and yeast strain YM4271 were included in the Matchmaker One Hybrid system kit (CLONTECH, Palo Alto, USA).

### Construction of an Activation Domain tagged cDNA-Library from *H. jecorina *by Directional cloning

For preparation of cDNA, mycelia of the wild-type strain QM9414, grown under cellulase inducing conditions (sophorose) were used. mRNA was isolated from total RNA using the PolyATtract mRNA Isolation Kit (Promega, Madison, USA), and a cDNA library was constructed using the HybriZAP™ Two-Hybrid cDNA Gigapack^® ^Cloning Kit (Stratagene Ltd., Cambridge, UK) according to the manufacturers instructions. After packaging by the Gigapack III Gold packaging extract (Stratagene Ltd, Cambridge, UK) and excision, the cDNA library containing 10^6 ^independent clones within the vector pAD-GAL4-2.1 in frame to the GAL4 activation domain was used in the One Hybrid System.

### Yeast One hybrid Screening of a *H. jecorina *cDNA Library

The yeast reporter strain HISLACZ-CAE was transformed using the lithium acetate/ssDNA/PEG method [37]. The resulting 3 × 10^5 ^yeast transformants were plated on his^-^/leu^- ^selective media containing 50 mM 3-aminotriazole (3-AT). The concentration of 50 mM 3-AT was previously determined to be sufficient to suppress leaky expression of the marker gene. Positive transformants were additionally tested for β-galactosidase activity by a filter lift assay according to the manufacturer's instructions (Matchmaker One-Hybrid system Kit, CLONTECH). Plasmids from putative positive clones were isolated after homogenization with glass beads, amplified in *E. coli *JM109 [38], and retransformed into yeast strain HISLACZ-CAE and tested for growth in the presence of 50 mM 3-AT. This step was followed by transformation of plasmids conferring histidine prototrophy into the negative control strain HIS-mCAE. The rationale was that the mutation abolishes protein binding in the yeast cell, and positive transformants should therefore be unable to grow on selective media.

### Cloning of chromosomal DNA of *H. jecorina*

To isolate the corresponding genomic clones, the cDNA fragment obtained from the experiment was used as probe to screen a genomic λ Blue Star (Novagen, Madison, Wis.) library of *H. jecorina *QM9414. Positive clones were excised from the phages by automatic subcloning following the instructions of the manufacturer. The sequence was crosschecked with the *Trichoderma reesei *Genome database v2.0 [39].

### 3' RACE

First strand synthesis was performed using the Reverse Transcription System (Promega, Madison, USA) according to the manufacturers protocol at 42°C for 1 hour with primer RACE-N (5' GCGTAATACGACTCACTATAGGGCGAATTGGGTTTTTTTTTTTTTTTTT(AGC) 3'). The reaction mixture was cleaned up using the QIAquick PCR Cleanup Kit (QIAGEN). PCR and Nested PCR were performed according to standard protocols using a gene specific primer and primer RACE-N22 (5' GCGTAATACGACTCACTAT AGG 3').

### Determination of biomass formation from cellulose grown cultures

After 96 hours of growth on microcrystalline cellulose either in light or in darkness, mycelia were harvested by centrifugation and 5 ml 0.1 N NaOH were added to the pellet. The sample was sonicated for 3 minutes and incubated for 3 hours at room temperature. Following centrifugation, protein content of the supernatant representing biomass content of the sample was determined using the BIO-RAD Protein assay (BIO-RAD, Hercules, US). Samples were analyzed in triplicates from independent cultivations.

### Purification of glutathione S-transferase fusion proteins

The cDNA-insert encoding aa_457 _– aa_636 _of LIM1 as isolated with the One Hybrid system was cloned into vector pGEX4T-1 (Stratagene). *E. coli *BL21 (Stratagene) was transformed with the expression construct. Expression and purification by glutathione sepharose 4B (Amersham Pharmacia Biotech, Uppsala, Sweden) affinity chromatography was essentially performed as described previously [40]. Fusion proteins were eluted using 50 mM Tris-HCl, pH 8.0, 10 mM glutathione and 2 mM dithiothreitol. All protein preparations were stored at -80°C in the presence of 20% (w/v) glycerol.

### Electrophoretic mobility shift assay

Electrophoretic mobility shift assays were performed as described by Stangl et al[41]. The oligonucleotides were end-labelled with (α-^32^P)-dCTP, using Sequenase version 2.0 (Amersham, Little Chalfont, UK). After purification by nondenaturing polyacrylamide gel electrophoresis binding was achieved by incubating 5 μg of protein with 5 ng of labelled fragment at room temperature for 10 min. GST-elution buffer (see above) containing 20% glycerol and lacking protein was used as negative control (sample referred to as free probe).

### Phylogenetic analyses

Amino acid sequences were aligned with Clustal X 1.81 [42] and then visually adjusted. Phylogenetic analyses were performed in MEGA 2.1 using the Minimum Evolution (ME) approach. Reliability of nodes was estimated by ME bootstrap percentages (BPME) [43] obtained after 500 pseudo-replications.

## Results

### Identification of a protein that binds to CAE by the Yeast One Hybrid System

We applied the yeast one hybrid system to identify proteins binding to CAE [23], as this approach allows to screen for DNA-protein interaction *in vivo *and thus bypasses common drawbacks of *in vitro *experiments such as altered binding characteristics caused by the experimental conditions or problems with the renaturation of the proteins *in vitro *[44-46].

**Figure 1:**
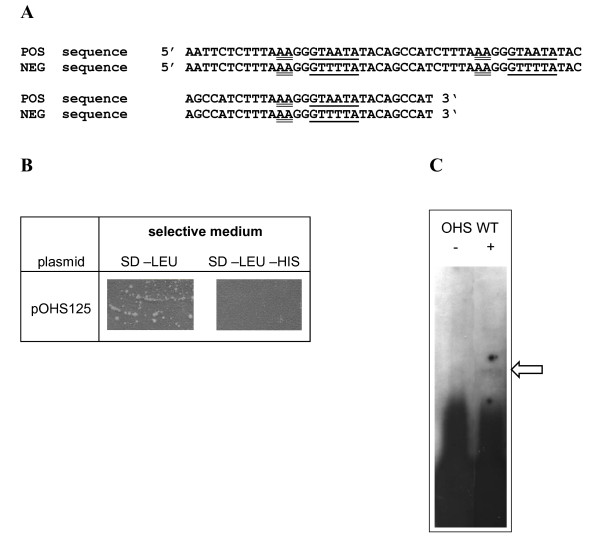
**Identification of the CAE-binding protein by the One Hybrid System**. (A) Sequences of oligonucleotides used for the in vivo yeast assay (POS; (GG)GTAATA; used to construct pHIS-CAE) and the negative control experiment (NEG (GG)GTTTTA; used to construct pHIS-mCAE). (B) Negative control experiment using pHIS-mCAE containing yeast strains transformed with pOHS125. Growth on synthetical dropout medium lacking leucine (SD-LEU; numerous colonies) indicates successful transformation, inability to grow on SD-LEU-HIS (empty plate, no colonies) confirms that due to the mutation of (GG)GTAATA to (GG)GTTTTA DNA binding of the protein encoded on pOHS125 is abolished and therefore sequence specific. A detail of the plates after the transformation is shown, equal amounts of transformation mix were plated on both positive and negative selection medium. (C) Electrophoretic mobility shift assay using 5 ng of labeled oneH-CAE as oligonucleotide and 5 μg of a GST-fusion protein with LIMPET aa_457 _– aa_636 _(+) which was functional in yeast. GST-elution buffer (see Materials and Methods) containing 20% glycerol and lacking protein was used as negative control (-; free probe). The complex formed is indicated by an arrow.

We used a sequence comprising a CCAAT to CCTTT – mutated CAE motif in order to prevent isolation of HAP-complex proteins, which bind to this motif [24] as bait (Figure 1A). Application of the One Hybrid System and subsequent screening for β-galactosidase activity led to the isolation of several clones representing proteins putatively binding to the (GG)GTAATA motif. The respective, positive plasmids were tested by retransformation into both the reporter strain HIS-LACZ-CAE to confirm DNA binding and also the negative control strain HIS-mCAE, in which the binding sequence bait (GG)GTAATA had been replaced by (GG)GTTTTA (Figure 1A). The latter mutation has previously been shown to abolish binding of proteins from cell free extracts of *H. jecorina *to CAE *in vitro *and result in lack of *cbh2*-gene expression *in vivo *[23]. In this control experiment, positive clones showed specific interaction to the wild-type bait (i. e. growth on selective medium) and no interaction with the mutated bait (i.e. no growth on selective medium). Plasmid pOHS125A enabled growth upon transformation into the reporter strain on selective medium comparable to the transformation control, but not if transformed into the strain comprising the mutated CAE motif (Figure 1B). Hence binding of the protein encoded on pOHS125A to CAE was confirmed and the lack of growth upon transformation into the negative control strain showed that this interaction was specific and was not due to unspecific binding to the promotor outside the region of interest. pOHS125A, which contains a 1.057-bp cDNA insert encoding a putative WD-repeat domain protein, which – as expected – was in frame with the GAL4 activation domain of the vector, was chosen for further investigation.

### The protein encoded on pOHS125 also binds to CAE in vitro

Since the protein encoded on pOHS125 was cloned by its ability to bind to the CAE motif *in vivo *in the One Hybrid Assay, we wanted to confirm this result *in vitro *by Electrophoretic mobility shift assay. To this end, the cDNA-insert from pOHS125 (encoding aa_457 _– aa_636_, which was functional in yeast) was cloned in frame into pGEX4T-1, expressed in *E. coli *as GST-fusion protein, purified and used in gel retardation assays. The EMSA analysis indicated that the fusion protein actually binds to the target sequence on the oligonucleotide oneH-CAE, albeit the interaction was very weak (Figure 1C).

### LIMPET encodes a WD-Repeat/F-box protein

**Figure 2:**
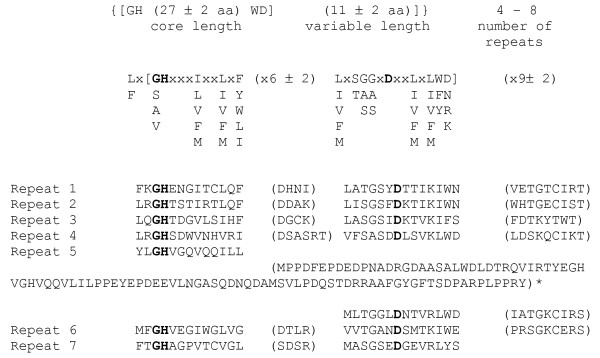
**Consensus sequence of WD40 repeats according to Neer et al., **[72]**and WD40 repeats of LIMPET**. (*represents an unusually long spacer region).

The 1.057-bp insert of pOHS125 was used to obtain a genomic clone and the 3' end as contained in pOHS125 was verified by 3'RACE. The predicted gene corresponds to the predicted protein tre77795 [39] and contains an open reading frame of 2004 bp interrupted by a 90 bp intron and encoding a 636 aa protein with a deduced molecular mass of 71.1 kDa. Despite its isolation on the basis of its ability to specifically bind to CAE, it lacks any of the known DNA-binding protein domains. A similar phenomenon was also reported earlier upon isolation of an SCF-component by the One Hybrid System [47]. Psort II analysis of the protein [48] revealed four nuclear localization sequences at amino acids 57, 186,196 and 239 and also predicted it as being localized to the nucleus according to Reinhardt's method for Cytoplasmatic/Nuclear discrimination [49]. A cAMP and cGMP dependent phosphorylation site at amino acid position 186 was found. No N-terminal signal sequence was detected. The C-terminal part of the protein contains 7 WD repeats (Figure 2) and the N-terminal part contains an F-box, a conserved motif first described by Kumar and Paietta [6]. Bai et al. [50] recognized that the F-box is a widespread motif required for protein-protein-interaction. A database search revealed that the encoded protein shows significant amino acid homology to several other WD-repeat/F-box containing proteins, highest identity and similarity among characterized proteins being obtained with SCON-2 of *Neurospora crassa *[51] (GenBank accession number Q01277; 58% and 71%, resp.), SconB of *Aspergillus nidulans *[5], (GenBank accession number Q00659; 57% and 72%, resp.) and *S. cerevisiae *Met30p (GenBank accession number NP_012218; 40% and 56%, resp.), all of them involved in the regulation of sulphur metabolism. Phylogenetic analysis of the 50 best hits in PSI-BLAST [52] revealed that it occurs at a basal position in a subclade containing other *Sordariomycete *proteins including SCON-2, which is part of a major, strongly supported clade also containing *A. nidulans *SconB (data not shown). The hierarchy of this tree is consistent with the present knowledge about evolution in the ascomycetes and therefore likely comprises the orthologues of SconB/SCON-2. Consequently, this protein is the *H. jecorina *orthologue of these two proteins. Analysis of the promotor revealed 4 GATA-type binding sites, whereas putative CYS-3 binding sites, which would be indicative of a control loop as present in *N. crassa *[6] are lacking. As the function of WD-domain/F-box proteins is in the attachment to other proteins, and to acknowledge its proposed function in cellulose signal transduction which has hot been reported from its closest orthologues, we have chosen to call this protein LIMPET (encoded by *lim1*).

### *lim1 *is regulated in dependence of sulphur availability

**Figure 3:**
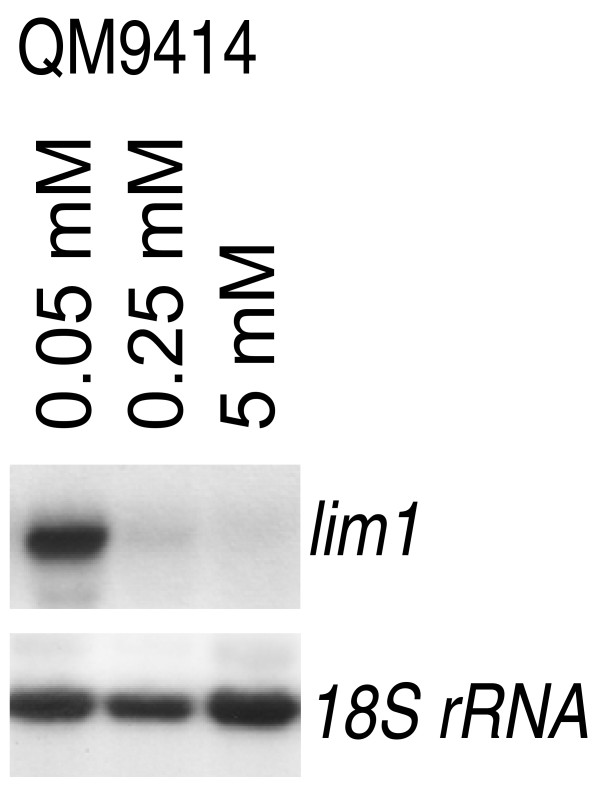
**Northern analysis of transcription of *lim1***. Strains were cultivated for 72 hours on Mandels Andreotti minimal medium lacking sulphate with 1% (w/v) glycerol as sole carbon source and supplemented with 0.05, 0.25 or 5 mM methionine as specified with the samples. 20 μg of total RNA were loaded per lane, a hybridization with 18S rRNA is given as a loading control.

Because of the *in silico *identification of LIMPET as a regulator of sulphur metabolism, regulation of *lim1 *was tested for its regulation under different levels of sulphur source availability. In order to ensure comparability to earlier studies we chose conditions similar to those used to investigate transcription of the respective orthologues in *Neurospora crassa *(SCON-2) or *Aspergillus nidulans *(SconB) [5,6]. Therefore *H. jecorina *QM9414 was cultivated on sulphate free Mandels Andreotti minimal medium in the presence of glycerol as sole carbon source supplemented with methionine as the only sulphur source at high (5 mM), low (0.25 mM) or limiting (0.05 mM) concentration. The data shown in Figure 3 indeed confirm that, as its counterparts in *Neurospora *and *Aspergillus*, *lim1 *is transcribed only on limiting concentrations of methionine in the medium, thus supporting the hypothesis that LIMPET is an orthologue of these two sulphur regulators.

### LIMPET responds to cellulase-inducing conditions

**Figure 4:**
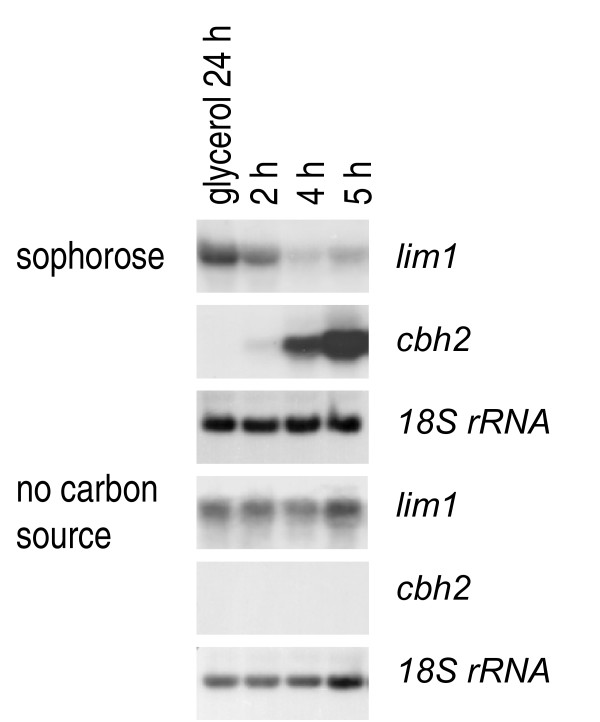
**Northern analysis of transcription of *lim1 *or *cbh2 *after induction of cellulase gene expression**. Cellulase gene expression was induced by replacement to medium containing 1.5 mM sophorose or medium lacking carbon source in constant darkness. Strains were grown on Mandels Andreotti minimal medium with 1% (w/v) glycerol as carbon source for 24 h in darkness (preculture). Mycelia were harvested 2, 4 or 5 hours after replacement. 20 μg of total RNA were loaded per lane, a hybridization with 18S rRNA is given as a loading control.

The isolation of LIMPET as a (potentially regulatory) protein binding to the cellulase activating element CAE suggests a function in induction and/or regulation of cellulase gene expression. Therefore we analyzed whether *lim1 *would transcriptionally react to a shift to inducing conditions. Again we chose conditions shown to strongly induce cellulase gene expression in earlier studies [23,31] and at the same time would reveal a short term response to inducing conditions. Strain QM9414 was grown on Mandels-Andreotti minimal medium with 1% (w/v) glycerol as sole carbon source for 24 hours in darkness and afterwards transferred to medium lacking the carbon source but supplemented with 1.5 mM (0.05%) sophorose, which has a strong cellulase inducing effect [30]. Under these conditions sophorose cannot be regarded as carbon source due to the extremely low amount, but only as an inducing agent for cellulase expression. Therefore the same medium without sophorose was used as control. In the presence of sophorose, transcript abundance after the shift to inducing conditions decreases as cellulase gene transcription increases (Figure 4). This effect was not due to the transfer to the new medium, because with the medium lacking sophorose neither the transcript abundance of *lim1 *decreases nor transcription of *cbh2 *is induced. Thus *lim1 *transcriptionally reacts to the presence of the strong inducer sophorose i. e. cellulase inducing conditions in the opposite way as cellulase genes. These data are also consistent with the hypothesis that LIMPET could act as a negative regulator of cellulase formation.

### Sulphate is important for normal growth of *H. jecorina *in light on cellulose

**Figure 5:**
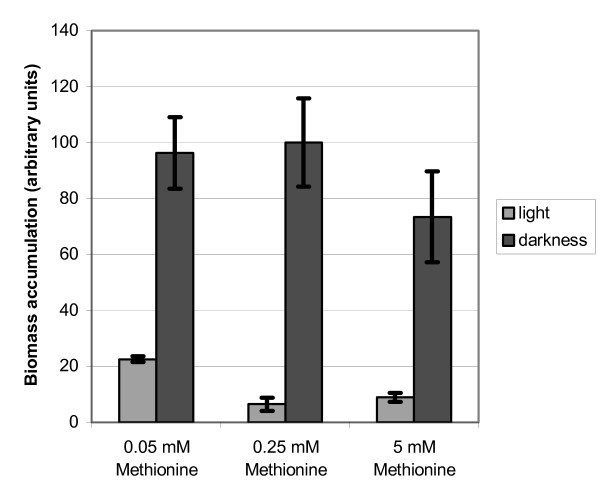
**Biomass formation of wild-type strain QM9414 in the presence of different sulphur concentrations**. Biomass accumulation was measured after growth for 96 hours in either complete darkness or constant light (1800 lux, 25 μmol photons m^-2^s^-1^) on sulphate free Mandels-Andreotti medium with 1% (w/v) of microcrystalline cellulose as carbon source supplemented with 0.05, 0.25 or 5 mM methionine. Values were normalized to growth on 0.25 mM methionine in constant darkness.

The experiments described above suggest a function in both sulphur metabolism and cellulase gene expression. The latter has recently been shown to be influenced by light [21]. Consequently we analyzed whether growth of *H. jecorina *on cellulose would be influenced by the availability of the sulphur source and whether the light status would be important for this effect. In order to provide defined conditions with respect to sulphur availability the medium was altered according to [5]. The *H. jecorina *QM9414 was grown for 96 hours on sulphate free Mandels Andreotti medium with 1% (w/v) microcrystalline cellulose as carbon source and methionine as the only sulphur source in high (5 mM), low (0.25 mM) or limiting (0.05 mM) concentration in light and darkness. We observed a reduction of biomass formation in light of 80 to 95% as compared to growth in constant darkness was observed (Figure 5). Although significant changes in biomass formation due to different amounts of methionine in the medium were observed, the concentration of supplemented methionine as sulphur source could not compensate the growth defect in light and also had no major effect on growth in darkness. Under commonly used conditions, i.e. the conventional Mandels Andreotti medium (which contains approximately 12 mM sulphate) corresponding to high sulphate concentration, *H. jecorina *only shows a decrease in biomass formation in liquid culture in light compared to growth in darkness of roughly 20% [21] after 96 hours of growth with microcrystalline cellulose as carbon source. Since the major difference of the two media used is their content of inorganic sulphate, which is lacking completely and replaced by different concentrations of the organic sulphur source methionine in one case, a role of inorganic sulphate as signal in processes related to light response seems possible. Nevertheless, an influence of the decreased amino acid availability in the sulphate free medium because of the lack of peptone as compared to the conventional Mandels Andreotti medium cannot be excluded, but does not explain the huge difference in growth between darkness and light under otherwise equal conditions.

### Sulphate uptake is regulated by light in *H. jecorina*

The finding that light-dependent biomass formation of *H. jecorina *on cellulose is clearly altered upon growth in medium lacking sulphate led to the hypothesis that uptake of inorganic sulphur is important for growth on cellulosic substrates in light. This hypothesis was tested in the following. Sensitivity versus toxic sulphur analogons such as selenate and chromate has been reported under conditions of low organic sulphur in wild-type strains of *Aspergillus nidulans *as well as *Neurospora crassa *[5,51]. This phenomenon is due to derepression of the sulphate uptake, when the organic sulphur source becomes limiting [53]. Because of the chemical analogy of selenate to sulphate, both are taken up into the cell by the same permeases [53,54] and also metabolism of selenate follows the same route as for sulphate [55]. The toxicity of selenate is consequently due to toxic by-products generated during reduction of selenate, which cause oxidative damage of DNA and addition of sulphate alleviates the toxicity of selenate [54,56]. Hence growth defects upon cultivation in the presence of selenate are indicative of selenate uptake into the cell. Considering the co-transport of sulphate and selenate such a growth defect also represents initiated sulphate uptake under the respective conditions.

Using plate experiments the presence of light-dependent regulation of sulphate uptake should be verified. Therefore plates containing sulphate free Mandels Andreotti medium with 1% (w/v) of (soluble) carboxymethylcellulose, 0.05 mM of methionine and either 1 mM of Na_2_SeO_4 _or 2 mM of Na_2_SO_4 _were inoculated with agar plugs taken from fully sporulated plates of strain QM9414. Afterwards they were exposed to light or kept in constant darkness under otherwise equal conditions at 28°C for four days.

In the presence of 0.05 mM (limiting) methionine Na_2_SeO_4 _was supposed to inhibit the growth of the fungus due to sulphur metabolite derepression [5]. In fact *H. jecorina *was not inhibited as severely as expected in growth by the presence of selenate and limiting concentrations of methionine. The observed colony diameter after four days of incubation of strain QM9414 was only slightly decreased on selenate compared to sulphate in constant darkness (2.7 ± 0.1 cm on selenate or 3.0 ± 0.1 cm on sulphate) i. e. by 10%, but was clearly reduced in light (2.5 ± 0.3 cm on selenate or 3.8 ± 0.2 cm on sulphate) i. e. by 35%. No such substantial light-dependent difference in selenate sensitivity was observed when the same experiment was done using glucose as carbon source under otherwise equal conditions (5.2 ± 0.4 cm on selenate or 6.2 ± 0.1 cm on sulphate in darkness (16%) and 4.9 ± 0.3 cm on selenate or 5.5 ± 0.4 cm on sulphate in light (12%)). Interestingly, increased concentrations of selenate caused only a minor decrease in growth up to 3 mM and no further decrease even with 10 mM selenate (data not shown), which completely inhibits growth of *Aspergillus *and *Neurospora*. The conclusion that sulphate uptake is increased during growth on carboxymethylcellulose in light (and therefore inhibited by the presence of selenate) is in perfect agreement with the data of biomass formation on microcrystalline cellulose as described above.

### *H. jecorina *lacks a homologue of the predominantly mycelial sulphate permease

**Figure 6:**
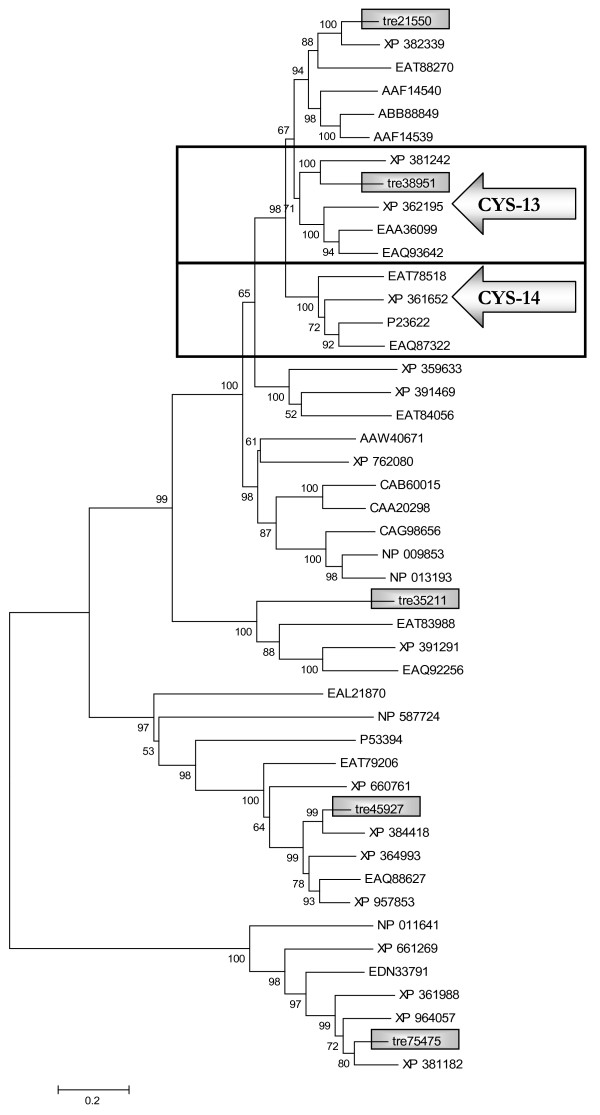
**Phylogenetic analysis of sulphate permeases of *H. jecorina***. Sulphate permeases present in the genome of *H. jecorina *and their nearest neighbours were analyzed with the software MEGA 4 using the Minimum evolution method. Description and GenBank accession numbers of the proteins used are listed in Table 1. *H. jecorina *sulphate permeases are provided with the proteinID as given in the *T. reesei *genome database v2.0. Numbers at branches indicate their boostrap support. Clusters comprising homologues of *N. crassa *CYS-13 and CYS-14, respectively, are boxed.

After the finding of an obviously altered regulation of sulphate uptake in *H. jecorina *as compared to *Neurospora *or *Aspergillus *we wondered whether this alteration could be due to a different machinery available for regulation of sulphate metabolism in this fungus. Therefore we screened the *Trichoderma reesei *genome database v2.0 [39] for genes with similarity to the four sulphate permeases detected within the *N. crassa *genome [57]. Phylogenetic analysis of the five proteins (tre21550, tre38951, tre35211, tre45927 and tre75475) we identified, revealed that only three of them clustered with the sulphate permeases present in *N. crassa *(Figure 6, Table 1). *N. crassa *CYS-14 [53,58], the predominantly mycelial sulphate permease, seems to have no orthologue in *H. jecorina*. Only an orthologue of the second characterized, predominantly conidial sulphate permease, CYS-13 [53] is also present in *H. jecorina *(tre38951). This sulphate permease shares 49% amino acid identity to the *N. crassa *hypothetical protein NCU03235.1 (XP_965335) which was denominated CYS-13 [57]. Since deletion of CYS-13 and CYS-14 leads to the inability to use sulphate for growth in other organisms [53], the remaining sulphate permeases are likely to have specialized functions and are not primarily involved in sulphate uptake under the conditions studied so far. Analysis of transcription of *H. jecorina cys13 *did not indicate expression of this sulphate permease under any conditions tested, including growth on glucose, glycerol or lactose (data not shown). Thus we conclude that *cys13 *does not compensate for the lack of *cys14 *and that uptake of sulphate in this fungus is altered as compared to *Neurospora *or *Aspergillus*.

**Table 1:** Sequences used for phylogenetic analysis of sulphate permeases

**Accession No.**	**Description**
AAA33615	*Neurospora crassa *sulfate permease II cys-14
AAB67550	*Saccharomyces cerevisiae *Sul2p: high affinity sulfate permease
AAF14539	*Penicillium chrysogenum *sulphate permease SutB
AAF14540	*Penicillium chrysogenum *sulphate permease SutA
AAw40671	*Cryptococcus neoformans *sulfate transporter, putative
ABB88849	*Aspergillus nidulans *sulphate permease SUL1
CAA20298	*Schizosaccharomyces pombe *sulphate permease SPBC3H7.02
CAA84506	*Saccharomyces cerevisiae *sulfate permease.
CAB60015	*Schizosaccharomyces pombe *sulphate permease SPAC869.05c
CAG98656	*Kluyveromyces lactis *unnamed protein product
EAA28274	*Neurospora crassa *sulphate permease NCU04433.1
EAA36099	*Neurospora crassa *hypothetical protein NCU03235.1
EAL21870	*Cryptococcus neoformans *hypothetical protein CNBC0120
EAL21871	*Cryptococcus neoformans *hypothetical protein CNBC0120
EAL23415	*Cryptococcus neoformans *hypothetical protein CNBA0650
EAQ71607	*Magnaporthe grisea *hypothetical protein MGG_ch7g1014
EAQ87322	*Chaetomium globosum *hypothetical protein CHGG_03941
EAQ88627	*Chaetomium globosum *hypothetical protein CHGG_05246
EAQ92256	*Chaetomium globosum *hypothetical protein CHGG_00491
EAQ93642	*Chaetomium globosum *hypothetical protein CHGG_01877
EAT78518	*Phaeosphaeria nodorum *hypothetical protein SNOG_14281
EAT79206	*Phaeosphaeria nodorum *hypothetical protein SNOG_13322
EAT83988	*Phaeosphaeria nodorum *hypothetical protein SNOG_08820
EAT84056	*Phaeosphaeria nodorum *hypothetical protein SNOG_08888
EAT88270	*Phaeophaeria nodorum *hypothetical protein SNOG_04510
EDN33791	*Botryotinia fuckeliana *hypothetical protein BC1G_12094
NP_009853	*Saccharomyces cerevisiae *sulphate permease Sul1p
NP_011641	*Saccharomyces cerevisiae *putative protein of unknown function
NP_013193	*Saccharomyces cerevisiae *sulphate permease Sul2p
NP_587724	*Schizosaccharomyces pombe *hypothetical protein SPCC320.05
P23622	*Neurospora crassa *sulphate permease 2 CYS-14
P53394	*Saccharomyces cerevisiae *Putative sulfate transporter YPR003C
tre21550	*H. jecorina *sulphate permease
tre35211	*H. jecorina *sulphate permease
tre38951	*H. jecorina *sulphate permease
tre45927	*H. jecorina *sulphate permease
tre75475	*H. jecorina *sulphate permease
XP_359633	*Magnaporthe grisea *hypothetical protein MG05144.4
XP_361652	*Magnaporthe grisea *hypothetical protein MG04126.4
XP_361988	*Magnaporthe grisea *hypothetical protein MGG_04433
XP_362195	*Magnaporthe grisea *hypothetical protein MG04640.4
XP_364993	*Magnaporthe grisea *hypothetical protein MG09838.4
XP_366031	*Magnaporthe grisea *hypothetical protein MG10251.4
XP_381182	*Gibberella zeae *hypothetical protein FG01006.1
XP_381242	*Gibberella zeae *hypothetical protein FG01066.1
XP_382339	*Gibberella zeae *hypothetical protein FG02163.1
XP_384418	*Gibberella zeae *hypothetical protein FG04242.1
XP_391291	*Gibberella zeae *hypothetical protein FG11115.1
XP_391469	*Gibberella zeae *hypothetical protein FG11293.1
XP_660761	*Aspergillus nidulans *hypothetical protein AN3157.2
XP_661269	*Aspergillus nidulans *hypothetical protein AN3665.2
XP_762080	*Ustilago maydis *hypothetical protein UM05933.1
XP_957853	*Neurospora crassa *hypothetical protein NCU09642.1

### Expression of *lim1 *and cellulase genes is dependent on the availability of the sulphur source

**Figure 7:**
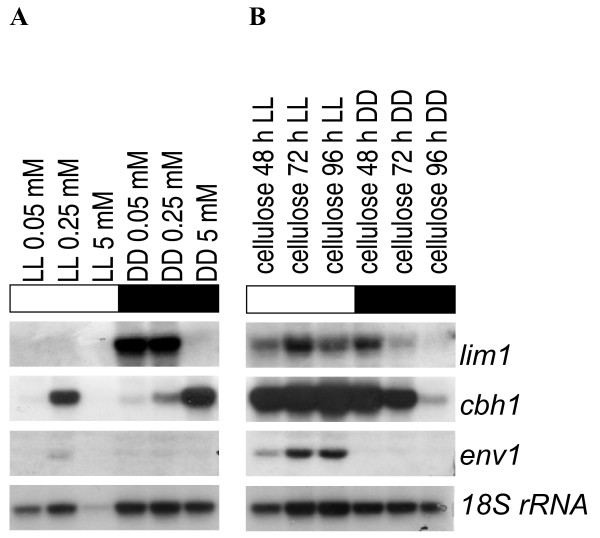
**Northern analysis of transcription of *lim1*, *cbh1 *and *env1 *in constant light (LL; 1800 lux, 25 μmol photons m^-2^s^-1^) or constant darkness (DD)**. (A) Strains were cultivated for 72 hours on Mandels Andreotti minimal medium lacking sulphate with 1% (w/v) microcrystalline cellulose as sole carbon source and supplemented with 0.05, 0.25 or 5 mM methionine as specified with the samples. (B) Strains were cultivated for the time indicated with the samples on conventional Mandels Andreotti minimal medium with 1% (w/v) microcrystalline cellulose as sole carbon source, which comprises approximately 12 mM sulphate and 0.25 mM methionine due to the addition of peptone. 20 μg of total RNA were loaded per lane, a hybridization with *18S rRNA *is given as a loading control.

The data described above suggests that availability as well as quality of the sulphur source are important for regulation of cellulase gene transcription and that the newly identified regulator protein LIMPET is involved in this process. If this would prove correct, then cellulase gene transcription should be influenced by the sulphur source in the medium and a correlation to *lim1 *transcription should occur. Since we detected a general influence of the sulphur source on growth on cellulose, we analyzed transcription of the main cellulase *cbh1*. Also the promotor of *cbh1 *comprises a motif similar to the binding sequence analyzed in this study at -783 relative to the ATG. As cellulases are coregulated in *H. jecorina *[20,59,60], transcription of *cbh1 *can be considered representative for cellulase transcription. Transcript abundance of both *lim1 *and *cbh1 *was analyzed upon growth in sulphate free medium supplemented with 0.05 (limiting), 0.25 (low) or 5 mM (high) of methionine in constant light or constant darkness. With methionine as the sole sulphur source, *lim1 *was transcribed in the presence of low or limiting (0.05 and 0.25 mM) concentrations and in darkness only. The transcription of *cbh1 *was regulated contrarily to *lim1 *being maximal at 5 mM methionine in darkness thus indicating a stimulation by methionine (Figure 7A). In light no *lim1 *transcript was detectable, and cellulase transcript abundance in the presence of 0.25 mM methionine was clearly elevated as compared to darkness, which is in concordance with earlier data [21]. However, no cellulase transcript was detected under sulphur limiting conditions in light. In the presence of 5 mM methionine growth in light was severely reduced, which did not allow for a reliable result for this condition.

Due to the obvious indirect correlation of transcription of *lim1 *and *cbh1 *in darkness, we were interested whether this correlation would also appear under commonly used conditions i. e. in conventional Mandels Andreotti medium, which contains 10 g/l peptone (Merck, #1.07213; corresponding to 0.25 mM methionine) and approx. 12 mM sulphate. Upon growth on conventional Mandels Andreotti medium, *lim1 *was detected not only in darkness, but also during growth in light. The complementary regulation of *lim1 *and *cbh1 *as seen under controlled sulphur conditions and upon sophorose-induction was not obvious in conventional Mandels Andreotti medium (Figure 7B). Since no inorganic sulphate is available in the sulphate free Mandels Andreotti minimal medium, it seems likely that *lim1 *responds to the kind of sulphur source available or even specifically to the presence of methionine if sulphate is lacking both by an altered transcript level in general as well as by an altered response to light.

### *lim1 *shows a transcriptional response to light and is regulated by ENVOY

**Figure 8:**
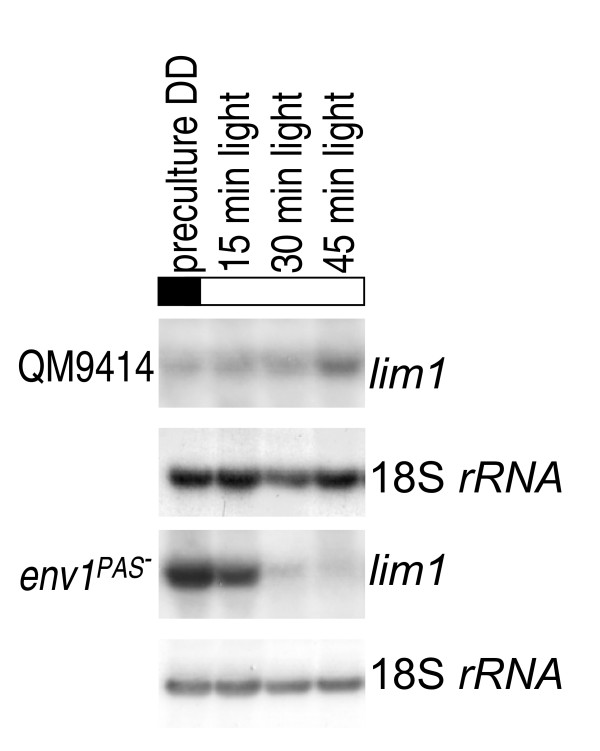
**Northern analysis of transcription for analysis of light response of *lim1***. Transcription was analyzed in constant darkness (preculture, DD) and after illumination (1800 lux, 25 μmol photons m^-2^s^-1^) for 15, 30 or 45 minutes in the wild-type strain QM9414 and a strain lacking the PAS-domain of the light regulatory protein ENVOY (Schmoll et al., 2005). Strains were cultivated on conventional Mandels Andreotti minimal medium with 1% (w/v) glycerol as carbon source. 20 μg of total RNA were loaded per lane, a hybridization with 18S rRNA is given as a loading control.

A light-dependent regulation of *lim1 *as indicated above would corroborate the obviously light dependent regulatory processes connected to sulphur metabolism. Therefore we were interested if transcription of *lim1 *would respond to illumination and if the light-regulatory protein ENVOY would be involved in the respective regulation. Upon growth on glucose *lim1 *showed enhanced transcription after a switch from constant darkness to constant light after 45 minutes on glucose. In the strain *env1*^PAS- ^lacking the PAS-domain of the light regulatory protein ENVOY [21], transcription of *lim1 *is strongly increased in darkness and decreases upon illumination (Figure 8). Therefore *lim1 *is at least under some conditions subject to regulation by ENVOY. These data are indicative of regulation of *lim1 *by light, but also indicate that *lim1 *falls into the category of genes repressed by ENVOY in the dark [22]. The *env1 *transcript itself is responsive to limiting methionine levels, since it is more abundant in the presence of 0.25 mM methionine as with only 0.05 mM methionine (Figure 7A).

### Cellulase gene transcription is influenced contrarily in light and darkness by methionine

**Figure 9:**
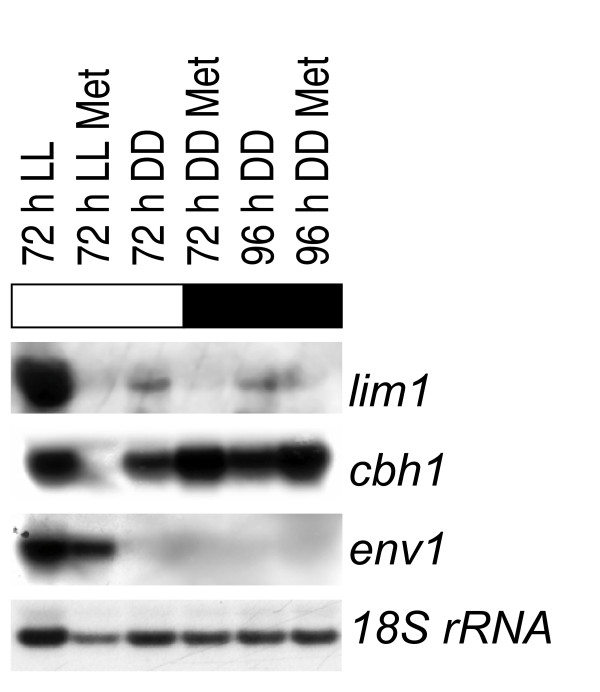
**Northern analysis of transcription of *lim1, cbh1 *and *env1 *in constant light (LL; 1800 lux, 25 μmol photons m^-2^s^-1^) or constant darkness (DD)**. Strains were cultivated for 72 or 96 hours on conventional Mandels Andreotti minimal medium with 1% (w/v) microcrystalline cellulose as sole carbon source. Met indicates addition of 5 mM methionine to the culture. 20 μg of total RNA were loaded per lane, a hybridization with 18S rRNA is given as a loading control. Transcript abundance was measured by densitometric scanning and normalization to the 18S rRNA loading control.

Since the increased cellulase transcription upon addition of 5 mM methionine in darkness (Figure 7A) could be exploited to enhance efficiency of industrial fermentations, we wanted to test whether this enhancing effect of methionine would also occur under conditions commonly used with *H. jecorina*. The fungus was therefore grown on Mandels Andreotti minimal medium with 1% (w/v) microcrystalline cellulose as carbon source and 0.1% peptone in light and darkness with or without 5 mM methionine. In darkness we observed a threefold increase in transcript abundance of *cbh1 *after 72 hours when methionine was added. In the presence of light, however, transcription of the major cellulase *cbh1 *was completely shut down under the same conditions (Figure 9), although in this case the severe growth inhibition as seen without sulphate was not observed. Therefore *H. jecorina *most probably utilized the carbon content of peptone for growth, which however does not induce cellulase gene expression (M. Schmoll, unpublished). In accordance with its function as sulphur metabolite repressor, *lim1 *responded both in light and darkness to increased methionine levels with a shutdown of transcription. Thus *lim1 *specifically reacts to methionine levels in the medium, but – assuming cellulase genes to be a target of LIMPET – the output of its regulatory function depends on the light status.

## Discussion

Sulphur uptake and metabolism are crucial processes for all organisms because of the manifold roles of the two sulphur containing amino acids cysteine and methionine not only in protein biosynthesis but also in redox potential homeostasis and methylation processes. Organisms have therefore developed sophisticated regulatory mechanisms to ensure controlled sulphur availability. As for fungi, the best analyzed machineries for this purpose are those of *N. crassa*, *A. nidulans *and particularly *S. cerevisiae*. In *H. jecorina *sulphur metabolism has not been studied so far.

The analysis of the influence of the sulphur source on regulation of cellulase gene expression was initiated by the unexpected finding, that LIMPET binds to a *cis*-regulatory motif of the *cbh2 *promotor. LIMPET is the first WD-repeat/F-box protein to be identified and characterized in *H. jecorina*. Concluding from the functions of its orthologues in other fungi, LIMPET could be a regulatory protein that activates transcription by phosphorylation-dependent degradation of a factor inhibiting expression of *cbh2*. Such a function would be in agreement with data from Zeilinger et al., [23], who showed that the complexes formed on CAE (the *cbh2 *activating element) are smaller under inducing conditions (sophorose), albeit the respective kinase and this probably repressing factor remain to be identified. Given the fact that E3 ubiquitin ligases themselves are frequently targets of degradation in an SCF-dependent manner [61,62], LIMPET could also itself represent this repressor. While deletion of the *lim1*-orthologues *scon2 *(*Neurospora *cr*as*s*a*) or *sconb *(*Aspergillus nidulans*) was successful, we were not able to obtain viable *lim1*-deletion mutants, what may be due to the obviously altered sulphur regulatory pathway. Interestingly, the *S. cerevisiae *orthologue of *lim1*, *met30 *is also an essential gene [8]. Nevertheless, we can therefore not be sure, whether LIMPET is indeed the transducer of the sulphur signal to the *cbh2*-promotor and thus a direct regulator of cellulase gene expression, although a connection between sulphur- and carbon-signaling became clearly obvious.

A hypothesis how LIMPET could impact cellulase gene expression can be deduced from the studies on regulation of sulphur metabolism in *N. crassa*: The regulatory cycle of SCON-2 and CYS-3 has been analyzed in detail in *N. crassa *and involves both regulation of transcription and activation of the respective proteins. During sulphur limitation, SCON-2 is present in high amounts, most probably in an inactive state. If the nutritional situation changes and an abundant sulphur source is encountered, this pool of SCON-2 can be rapidly activated and prevent the function of CYS-3, and thus shut down the sulphur circuit. This finally results in the termination of sulphur uptake. On the other hand expression of the *scon-2 *gene, the promotor of which contains several CYS-3 binding sites, is dependent on functional CYS-3 [6,63]. Thus, SCON-2 inhibits *cys3 *transcription in a negative feedback loop [7]. CYS-3 activates the uptake of sulphate and several other mechanisms involved in sulphur metabolism [7]. Our data show that *lim1 *is not transcribed in light under conditions lacking an inorganic sulphur source. A function of LIMPET putatively analogous to SCON2 would thus lead to stimulated uptake of sulphate in light, which is not available under the condition tested, obviously resulting in considerably decreased growth. Also the decreasing effect of selenate upon growth on carboxymethylcellulose in light is in agreement with these data. Hence we conclude that sulphate is important for normal growth in light on cellulose and that methionine, being commonly considered an organic sulphur source, cannot compensate for this requirement. However, we want to note that despite the presence of a CYS-3 homologue, no binding sites similar to those identified in *N. crassa *were detected in the promotor of *lim1*. Hence a negative feedback loop may not be operative in *H. jecorina*.

Although the finding of LIMPET as a close homologue of *N. crassa *SCON-2 or *A. nidulans *SCONB, respectively, suggests similarities in the sulphur regulatory systems of *Hypocrea*, *Neurospora *and *Aspergillus*, the mechanism of sulphate uptake in *Hypocrea jecorina *QM9414 appears to be altered: First, phylogenetic analysis of its predicted sulphate permeases indicates, that the predominantly mycelial sulphate permease (the CYS-14 orthologue) is missing. Transcription of the remaining, predominantly conidial sulphate permease (the CYS-13 orthologue) could not be detected under any conditions tested (M. Schmoll, data not shown). In this respect it is particularly interesting, that lack of the two sulphate permeases CYS-13 and CYS-14 leads to the inability to use sulphate as sulphur source and to selenate resistence [53] in *N. crassa*, although 4 sulphate permease genes have been detected in this fungus [57]. For both *N. crassa *and *A. nidulans *[5,51] sensitivity versus toxic sulphur analogues in presence of low concentrations (0.25 mM) of methionine was described, which is not found for *H. jecorina*. In *S. cerevisiae*, regulation of the synthesis of both sulphate permeases is under the control of exogenous methionine or S-adenosylmethionine. Their synthesis is coordinated with the synthesis of the other methionine biosynthetic enzymes [64], what would imply continued uptake of selenate and thus lethality under low-methionine conditions. Judging from this data, the *H. jecorina *sulphur assimilation system appears to exhibit high affinity versus methionine, enabling the fungus to survive even in the presence of very low concentrations of it. Since this fungus can also utilize sulphate as sole sulphur source, it is likely that *H. jecorina *is – obviously unlike *A. nidulans *or *N. crassa *– able to differentiate between these sulphur sources (which is also reflected by the transcription pattern of *lim1 *on media with or without sulphate) and terminate one uptake mechanism in favour of the other if required. In case of the presence of selenate such a flexible mechanism could prevent the fatal effect of this sulphate analogon, although therefore a specific detection of the toxin would be required. Alternatively an as yet unidentifed detoxification mechanism, which would explain this effect could be at work. In this respect it is interesting, that for several yeast species growing on decaying fruits comparable mechanisms have been detected and suggested to have evolved due to the considerable amounts of selenate present in certain plants. In order to avoid the harmful consequences of sulphate and thus selenate uptake they can preferentially utilize organic sulphur, in many cases obtained by attacking other yeasts [65,66]. Data on the role of the *S. cerevisiae *homologue of LIMPET, Met30p clearly show a role of the sulphur regulatory machinery in heavy metal response through regulation of the transcription factor Met4p [11]. The main target of this circuit in this case is glutathione synthesis, which is needed to complex and subsequently detoxify the heavy metal. A mechanism as described above would be in concordance with the symbiotic interaction of *Trichoderma *spp. with plants [67], their natural substrate being decaying plant material and their efficient biocontrol activity [68]. The fact that both the presence of selenate/sulphate or methionine, respectively, has different consequences in light and darkness further indicates that these environmental cues must have a more significant relevance than just signalling the availability of sulphur. In accordance with this hypothesis we found that *H. jecorina *adjusts cellulase gene transcription in dependence of the sulphur source available and the light status. Considering the mechanisms discussed above the connecting environmental cues might be the presence of plant material (cellulose) and possibly the encounter of a competitor, which is more likely on the surface of the substrate (light).

Consequently, an involvement of LIMPET in modulation of cellulase gene expression in response to a varying supply of sulphur seems to be beneficial for *H. jecorina*. Interestingly, the respective signal leads to opposite effects in light and darkness in the presence of sulphate. Therefore we hypothesize that the significance of the presence of methionine reaches beyond merely an indication of the sulphur source. Since the addition of methionine did not stimulate growth on cellulose in light in the absence of sulphate, we assume that its effect is also not simply related to the provision of a potentially growth-limiting amino acid. The response to methionine may be – at least in part – connected to the intracellular cAMP-levels which has been shown to stimulate cellulase formation in *H. jecorina *[69] and *Cryphonectria parasitica *[70]. While the enhanced transcription of *cbh1 *upon addition of methionine in darkness is well in concordance with this hypothesis, the abolished *cbh1 *transcription under the same conditions in light rather indicates a more sophisticated regulation. A recent study by Xue and coworkers [71] provides intriguing hints which might shed a new light on the interacting signal transduction pathways studied in this work. They show that the G-protein coupled receptor GPR4 of *Cryptococcus neoformans*, which has an orthologue in *H. jecorina *(tre72004), interacts with the G-protein α subunit GPA1, the orthologue of *H. jecorina *GNA3 and that methionine induces cAMP accumulation in this fungus. This study on the one hand shows that this G-protein coupled receptor does not respond to glucose as expected from its structural similarity to the *S. cerevisiae *glucose sensor GPR1, but instead seems to sense methionine – this amino acid being the only clearly confirmed ligand of GPR4. On the other hand, its interacting G-protein α subunit GPA1 is involved in glucose sensing. Also, methionine was found to induce cAMP accumulation in *C. neoformans*.

## Conclusion

**Figure 10:**
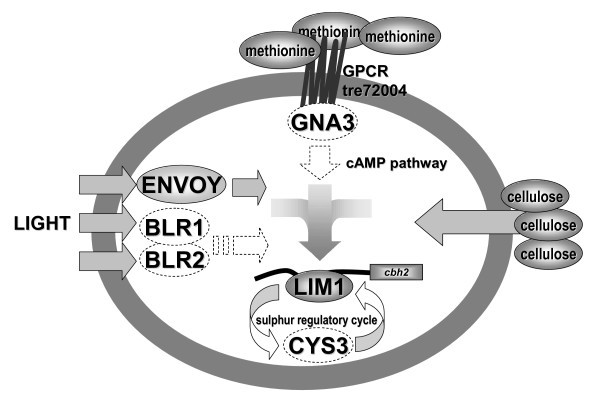
**Model of regulatory inputs on LIMPET and thus cellulase gene expression**. Environmental cues investigated in this study are represented in full, additional hypothetical components of the regulatory network as inferred by reports from other fungi are given as empty circles/arrows with dotted lines.

Out data revealed that cellulase gene expression is influenced by both quality and quantity of the sulphur source available and that the regulation of sulphur metabolism and -uptake in *H. jecorina *is different compared to other fungi. Thereby, the methionine signal seems not only to be representative for the presence of a sulphur source, but also has a light dependent significance for cellulase transcription. We hypothesize that *H. jecorina *GNA3, which we found to be involved in regulation of cellulase gene expression (manuscript submitted), could establish the connection between the putative methionine receptor and the target (Figure 10). Light dependent regulation of *lim1 *transcription in the presence of high amounts of inorganic sulphate and low methionine concentrations suggests that *lim1 *also responds to the kind of sulphur source available and might affect its targets accordingly. For SCON2 [51] and SCONB [5], although grown on glucose and without controlled light conditions, essentially similar transcriptional behaviour as observed with *lim1 *in darkness in the absence of sulphate, was reported. Therefore the function of *lim1 *is unlikely to be shifted from regulation of sulphate to regulation of carbon metabolism but lies rather in the connection of these pathways. Such a system would resemble a nutrient coincidence detection system as suggested by Xue and coworkers [71], which additionally integrates the light status into the regulation of transmission of the signal to its target.

## Authors' contributions

GG carried out the transcription analyses on cellulose and participated in drafting the manuscript. MD worked on deletion of *lim1 *and performed studies on selenate sensitivity. MS carried out the One Hybrid System, selection of positive clones, phylogenetic analyses, transcription analysis of response of *lim1 *to different sulphur conditions, cellulase inducing conditions and light response. MS conceived of the study and wrote the manuscript.
